# Uncovering different states of topological defects in schlieren textures of a nematic liquid crystal

**DOI:** 10.1038/s41598-017-16967-1

**Published:** 2017-12-01

**Authors:** Takuya Ohzono, Kaoru Katoh, Chenguang Wang, Aiko Fukazawa, Shigehiro Yamaguchi, Jun-ichi Fukuda

**Affiliations:** 1 Research Institute for Sustainable Chemistry, National Institute of Advanced Industrial Science and Technology (AIST) 1-1-1 Higashi, Tsukuba, 305-8565 Japan; 2 Biomedical Research Institute, AIST, 1-1-1 Higashi, Tsukuba, 305-8566 Japan; 3 Institute of Transformative Bio-Molecules (ITbM), Nagoya University, Furo, Chikusa, Nagoya, 464-8602 Japan; 4 Department of Chemistry, Graduate School of Science, and Integrated Research Consortium on Chemical Sciences (IRCCS), and Nagoya University, Furo, Chikusa, Nagoya, 464-8602 Japan; 5 Department of Physics, Kyushu University, 744 Motooka, Nishi-ku, Fukuoka, 819-0395 Japan

## Abstract

Topological defects are ubiquitously found in physical systems and therefore have been an important research subject of not only condensed matter physics but also cosmology. However, their fine structures remain elusive because of the microscopic scales involved. In the case of a liquid crystal, optical microscopy, although routinely used for the identification of liquid crystal phases and associated defects, does not have resolution high enough to distinguish fine structures of topological defects. Here we show that polarised and fluorescence microscopy, with the aid of numerical calculations on the orientational order and resulting image distortions, can uncover the structural states of topological defects with strength *m* =  ±1 in a thin cell of a nematic liquid crystal. Particularly, defects with *m* = +1 exhibit four different states arising from chiral symmetry breaking and up-down symmetry breaking. Our results demonstrate that optical microscopy is still a powerful tool to identify fine states of liquid crystalline defects.

## Introduction

Liquid crystals (LCs)^1–5^ have attracted interests of physicists as ideal model systems which facilitate direct observation of the structures of topological defects by optical means; for example, LCs have been studied as a tabletop model system of cosmic strings^6,7^. Not only from such a fundamental point of view, LCs defects have been drawing attention also as tuneable or reconfigurable templates for nano/micro-patterning of small non-LC objects, e.g., micro particles^8–15^, polymers^16,17^, self-assembled molecules^18,19^ and low molecular weight solutes^20,21^. Among different phases of LCs, the simplest nontrivial phase is the nematic phase characterised by broken rotational symmetry, while retaining the translational symmetry. Its local orientational order is specified by a unit vector without head-tail distinction, known as the director commonly denoted by ***n*** (ref.^1^).

**Figure 1:**
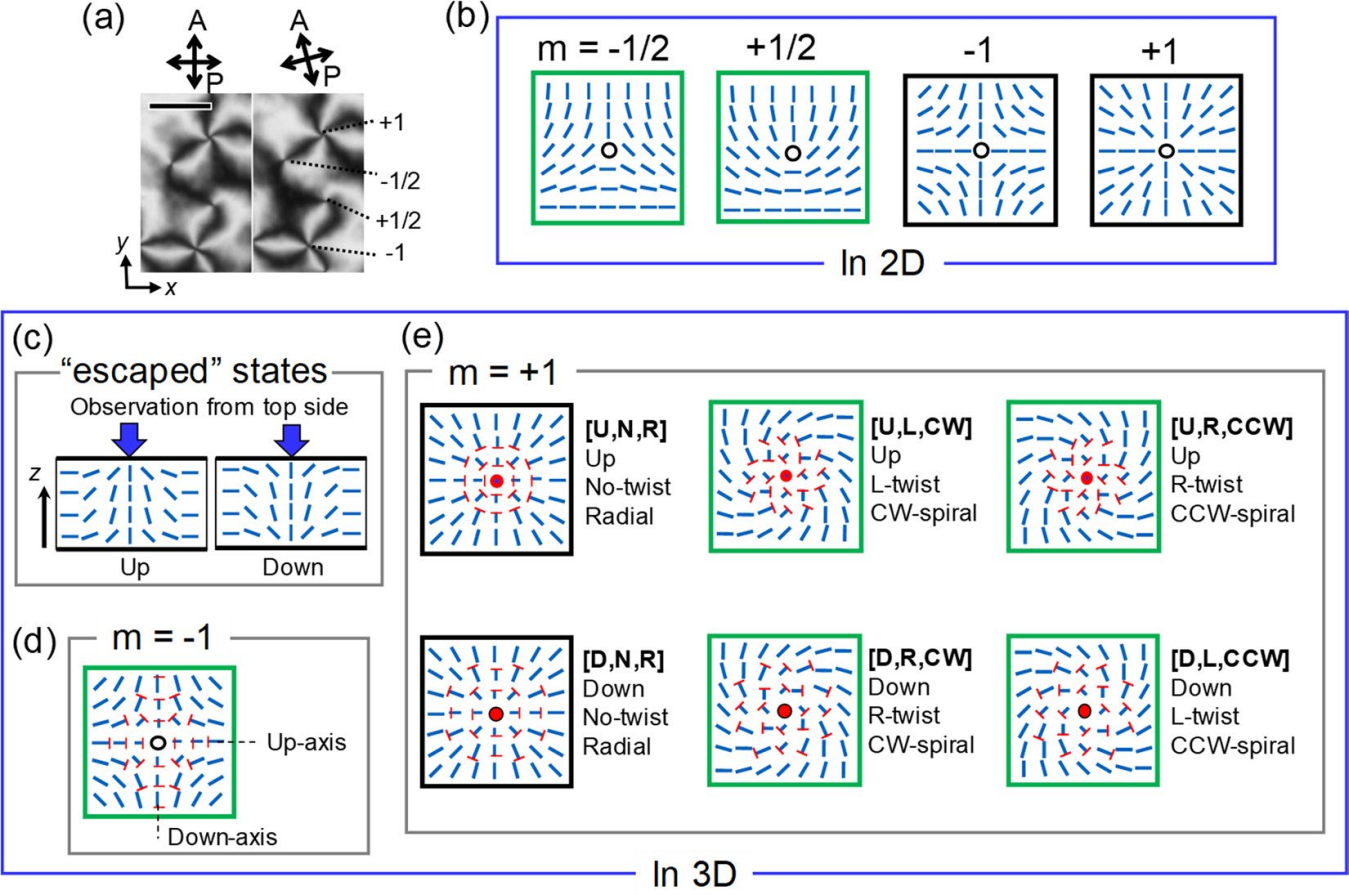
Types of topological defects in schlieren textures on a thin planar cell of NLCs. (**a**) A schlieren texture found under POM with crossed polarisers, showing four types of defects characterized by *m* =  ±1/2 and ±1. (Bar: 10 μm) (**b**) Schematics of typical defects when the director ***n*** is confined within two dimensions (2D). Blue sticks represent the distribution of ***n*** around the singularity indicated by circles. (**c**) Schematics of two representative cross sections of “escaped” structures for defects with integer *m* in a 3D system. Although they are identical when rotated about an axis perpendicular to the page, they are distinct when observed from one side of the LC cell. (**d**) Schematic of defect with *m* = −1 in 3D. The nail symbols indicate the projection of ***n*** onto the plane of the page (the red heads of the nails come out of the page, the positive direction of the *z* axis). The structure has two characteristic axes, shown as up-axis and down-axis, and it has C_2v_ symmetry with respect to the *z* axis. (**e**) Schematics of different states of the defect with *m* =  +1 in 3D with C_∞_ symmetry with respect to the *z* axis. The bold characters in square brackets represent distinct states; broken up-down symmetry (Up/Down), internal twist of ***n*** (No twist/Right-handed twist/Left-handed twist), and the appearance of the streamline of ***n*** from the centre in the plane (Radial/ClockWise spiral/Counter-ClockWise spiral). Two states on the left, [U,N,R] and [D,N,R], are the simplest “escaped” structure with pure splay and bend deformations without twist. Four states on the right, [U,L,CW], [U,R,CCW], [D,R,CW] and [D,L,CCW], are chiral-(or reflection)-symmetry-broken structures with twist deformations around defects. The six states framed by green squares are experimentally confirmed. When a chiral dopant (CB15) inducing right-handed twists are added, only [U,R,CCW] and [D,R,CW] should be observed.

The head-tail symmetry of ***n*** reflects the apolar nature of the nematic liquid crystal (NLC), and thus the order parameter space of a two-dimensional (2D) NLC is *S* ^1^/*Z*_2_, a circle (*S* ^1^) whose two ends of a diameter are identical. Topological structures of the order parameter space and associated defects are described by the homotopy groups of the order parameter space. The first homotopy group of *S* ^1^/*Z*_2_ is *Z* (refs^3,5^), that is, defects in a 2D NLC with different “winding number” *m* are topologically distinct. Moreover, *m* can be a half-integer as well as an integer because of the head-tail symmetry (Fig. 1b). The easiest way to determine *m* is an observation using a polarised optical microscope (POM). Under a POM, a NLC in a flat cell imposing planar surface alignment with no preferential direction (degenerate planar alignment) exhibits schlieren textures^22,23^ with topological defects from which dark brushes of even number (*k*) emanate (Fig. 1a). In a dark brush, ***n*** is parallel or perpendicular to the polariser (or analyser) axis, and the winding number *m* is related to *k* simply by *k* = 4|*m*|. The sign of *m* can be distinguished by the rotation of the crossed polarisers; dark brushes from a positive (negative) defect rotate in the direction the same as (opposite to) that of the polarisers.

The order parameter space of a 3D NLC is *S* ^2^/*Z*_2_, and its first homotopy group is *Z*_2_, different from that of a 2D NLC^3,5^. A direct and notable consequence of this difference is that a line defect is topologically stable only when its winding number *m* is half-integer; one with integer *m* can be smoothed out to a configuration with no singularity^1–5,24–30^. This is achieved by out-of-plane distributions of ***n***, often referred to as “escape to the third dimension” (Fig. 1c–e). This escape occurs naturally to relax the energetic cost of defect core regions, and a well-known example can be found in a cylindrical capillary imposing surface normal alignment^1,24–30^. Analogous structures known as umbilics can be induced by applying an electric field to a NLC with negative dielectric anisotropy^1,31^.

Although some of the 3D distributions of ***n*** in the regions surrounding specific defects in thick cells have been clarified with confocal fluorescence polarised microscopy^32–34^, fine defect structures in schlieren textures of a thin flat cell, especially those involving 3D profiles, remain unexplored experimentally because of the limitation in the resolution of optical microscopy. Moreover, POM does not provide direct information on the component of ***n*** normal to the cell, and therefore POM alone cannot capture the escaped configuration of the defects. Here, to uncover the structural states of defects, particularly those with *m* =  ±1, we present POM and fluorescence optical microscopy (FOM) observations of the defects. Complementary FOM using dyes with different fluorescence anisotropies can confirm the presence of escaped structures at the defects with *m* =  ±1. Combining experimental POM and FOM images with numerically calculated distributions of ***n*** and simple ray-tracing calculations to simulate the lensing effect due to the escaped structures, we uncover four different states of the defects with *m* =  +1, attributable to the up/down symmetry breaking of the escape, and also the chiral symmetry breaking of the distribution of ***n*** (Fig. 1e). Anisotropic configurations of the defects with *m* = −1 (refs^35,36^) are also identified (Fig. 1d). We show that the escaped structures cause different lensing effects depending on the direction of the escape and thus, different appearances of defects in the FOM images.

## Results

### Anisotropic fluorescence emissions of dyes in aligned NLCs

**Figure 2:**
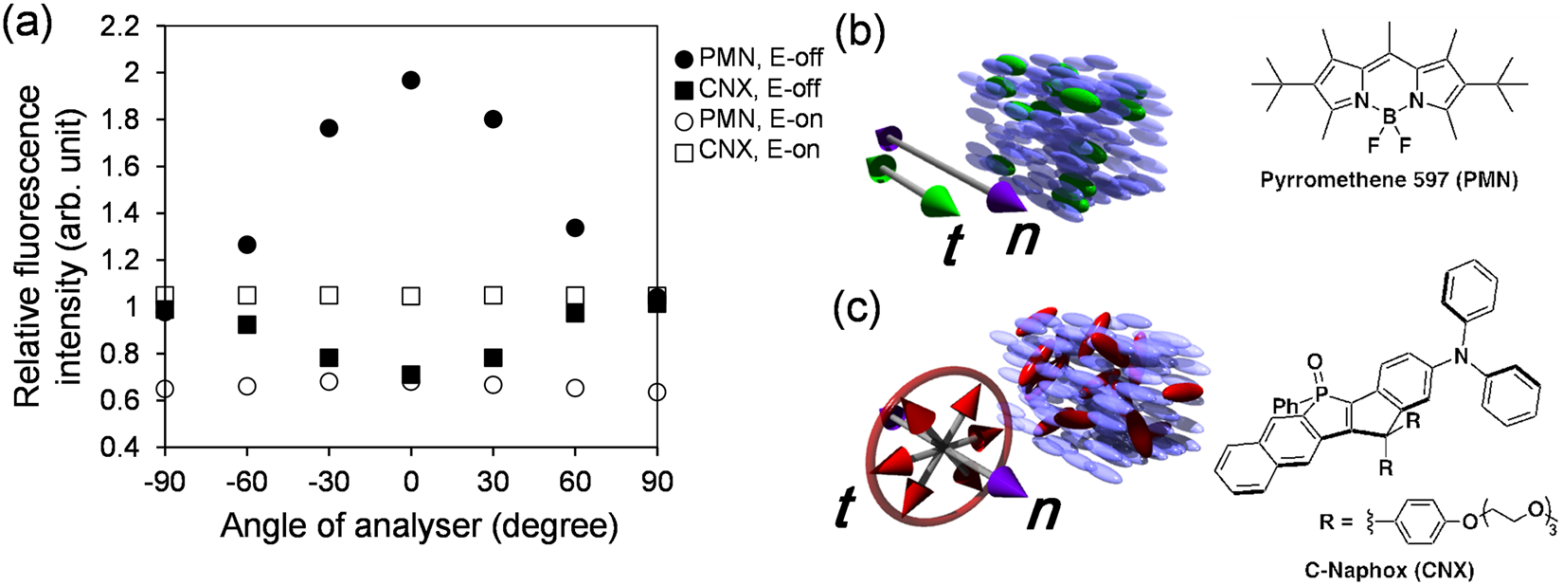
Positive and negative fluorescent dichroic properties of dyes in NLC. (**a**) Analyser-angle-dependence of relative fluorescence intensities of dye-doped NLCs in a planar cell with the thickness of 2 μm collected using the fluorescence microscopy setup. The analyser at 0 degree is parallel to the director ***n*** without an electric field. An AC electric field (peak-to-peak 10 V at 100 Hz) is applied in the surface normal direction to orient ***n*** perpendicularly. The relative fluorescence intensities are values normalized by those at the angle of ±90 degree without electric field. (**b**) and (**c**), Schematics of the average configurations of transition dipole moments, ***t***, of the dyes in NLCs (blue ellipsoids), along with their chemical structures. ***t*** of pyrromethene 597 (PMN) (shown by green colour) aligns along ***n***, indicating that PMN in NLC has positive dichroic property. ***t*** of C-Naphox (CNX) (shown by red colour) is distributed perpendicularly to ***n*** with rotational freedom in the plane normal to ***n***, indicating that CNX has negative dichroic property.

We first investigate the basic properties of the anisotropic fluorescence emissions from the dyes, pyrromethene 597 (PMN) and c-Naphox (CNX)^37^, in a uniaxially aligned NLC. Figure 2 shows the angle dependent fluorescence emissions with or without an electric field normal to the planar NLC cell. Without the electric field, both dyes show anisotropic emissions. The maximum emissions from PMN and CNX are found at the directions parallel and perpendicular to ***n*** = (*n*_*x*_, *n*_*y*_, *n*_*z*_), respectively. The results suggest that the transition dipole moment of the dye, ***t***, projected onto the cell plane lies along ***n*** for PMN and perpendicularly to ***n*** for CNX on average. With the electric field that aligns ***n*** normal to the cell plane, both dyes show isotropic emissions around the axis of ***n***. The results support that ***t*** indeed lies along ***n*** for PMN (positive fluorescence dichroic property) and perpendicularly to ***n*** for CNX (negative fluorescence dichroic property) on average (Fig. 2b,c).

The total fluorescence intensity without the analyser given by the integral of the plot of Fig. 2a over the angle is much lower under an electric field than without the field in the case of PMN. This suggests that in the FOM images of schlieren textures the region with the larger |*n*_*z*_| will appear darker. In the case of CNX, the relationship between the FOM intensity and |*n*_*z*_| is opposite to that of PMN; the region with larger |*n*_*z*_| will appear brighter. Therefore, using both dyes with positive and negative fluorescence dichroic properties, the distribution of |*n*_*z*_| in the schlieren textures can be qualitatively estimated in a complementary way. (Since the incident angle of the light through the objective lens is not perfectly parallel to the optical axis *z* in the FOM setup, the degree of polarization is not strictly maintained. Therefore, the present fluorescence dichroic properties should be regarded as qualitative.)

### Schlieren textures at different thicknesses

**Figure 3:**
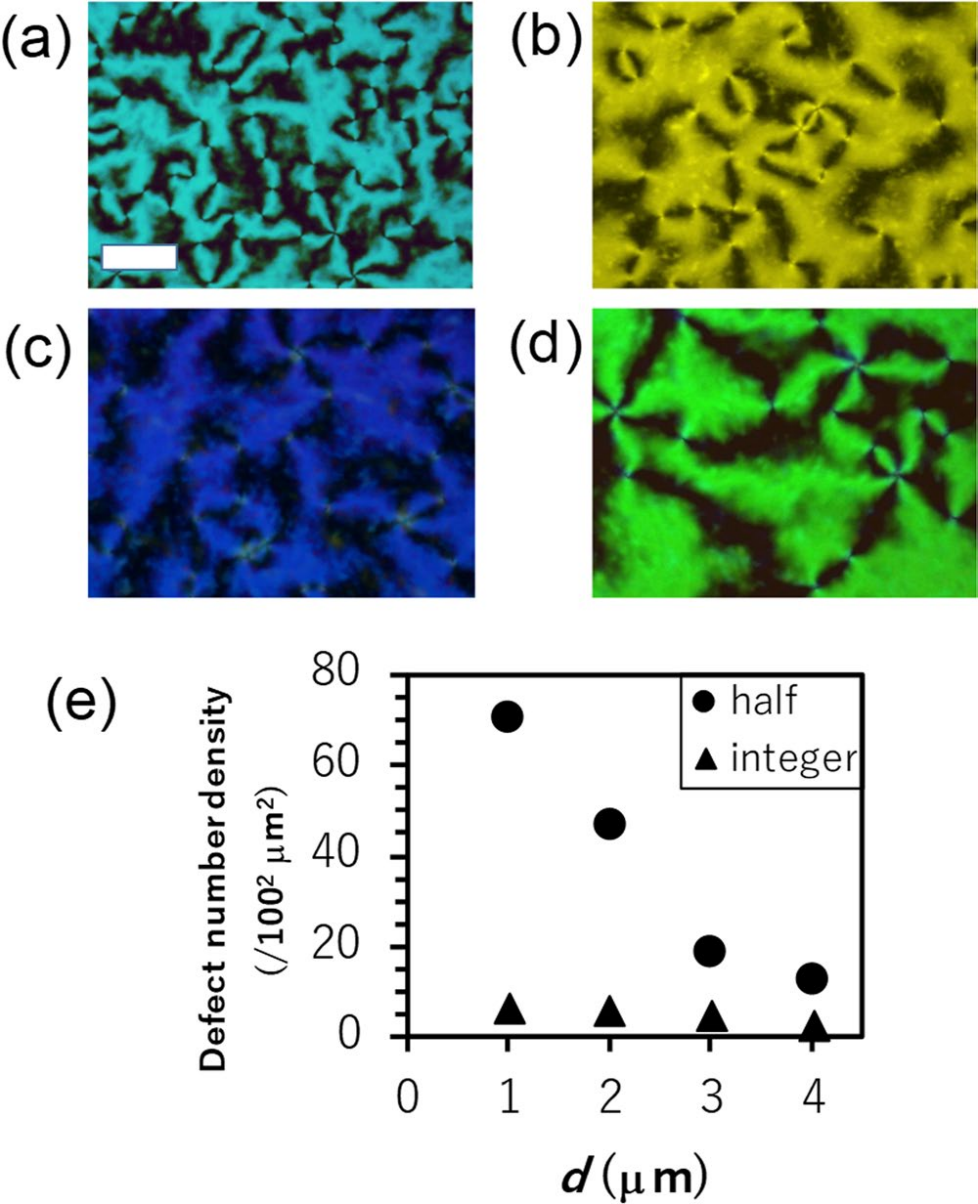
Four regions with different cell thickness showing typical schlieren textures. Schlieren textures at the approximate thickness *d* of (**a**) 1, (**b**) 2, (**c**) 3, and (**d**) 4 μm. The colours arise from the interference due to the thickness dependent retardation of the NLC (5CB) with planar alignment. (Bar: 20 μm) (**e**) Number densities of defects with half integer and integer *m* at different thicknesses.

Figure 3a–d shows typical schlieren textures observed at different local cell thicknesses *d* in a wedge cell (see Methods). The local thickness can be estimated by the colours arising from the thickness dependent retardation of the NLC (5CB) with planar alignment (see Methods). Four regions having local thickness of approximately 1, 2, 3, and 4 μm were analysed in the following analysis.

The number densities of defects with half-integer and integer *m* are plotted in Fig. 3e, showing decrease with increasing *d*. In general, a pair of defects with the same |*m*| and opposite signs tends to be annihilated after they attract each other^38–41^, which gives rise to coarsening of local nematic domains. In the present system, the transient and local alignment direction at the polyimide surfaces is memorized and frozen in short time scales (several seconds) after the liquid crystal is quenched to the nematic phase from the isotropic phase (surface memory effect^42–46^). Therefore, the observed frozen defects should be a consequence of the competition between the defect annihilation and the surface memorizing processes.

Since the surface memory grows only at the interface, the cell thickness should not affect the characteristic time scale of the growth. On the other hand, the coarsening process of local nematic domains that reduces the number of defects has more chances to proceed in a thicker cell. This may be a qualitative reason for the decrease in the total number of defects with increasing *d*. In contrast to the sharp decline of number density of defects with half-integer *m* with *d*, those with integer *m* decay more slowly. The defects with integer *m*, which emerge with the lower density, may have less chance to meet and be annihilated. Although the detail of the survival dynamics of defects is an interesting problem, it is beyond the scope of this paper. For the evaluation of the stable and frozen defects specifically with integer *m*, the present range of *d* is technically favourable because a sufficient number of defects for analysis can be acquired from single images captured at one time.

### Observation of defects in schlieren textures

**Figure 4:**
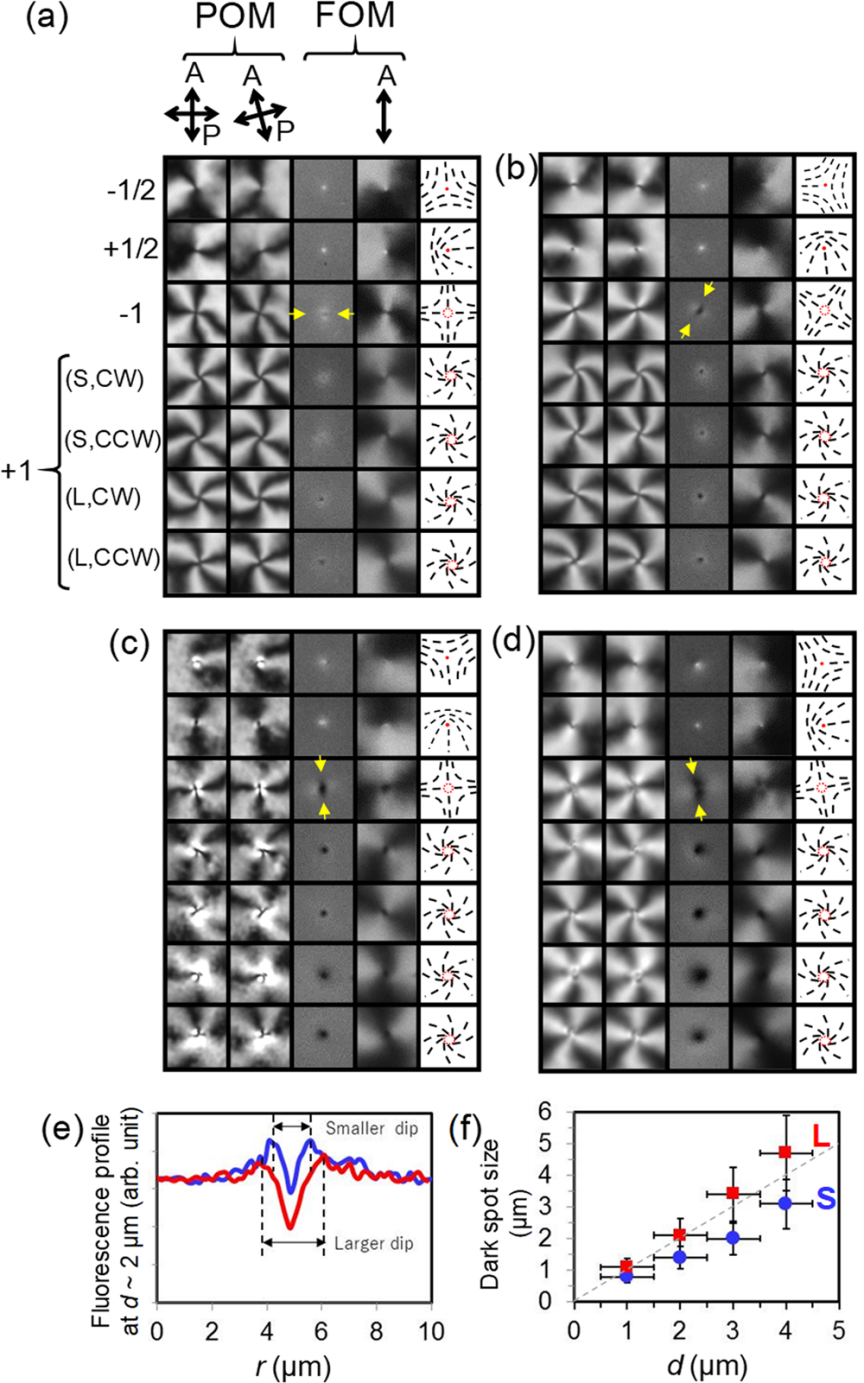
Discernible states of the defects found in schlieren textures of NLC doped with PMN. A set of microscope images (10 × 10 μm^2^ squares) of discernible defects with *m* =  ±1/2 and ±1. Shown are POM images with crossed polarisers, those with polarisers rotated by 15 degrees, FOM images and those with an analyser from left to right at *d* ~(**a**) 1, (**b**) 2, (**c**) 3, and (**d**) 4 μm. Corresponding schematics of the distribution of ***n*** projected onto the cell plane, in which black sticks represent local ***n***, are shown in each rightmost square. Yellow arrows indicate the direction of the elongation of dark spots for the defect with *m* = −1, which correspond to one of the characteristic axes of the defect with C_2v_ symmetry. An example of the original images with schlieren texture is shown in Supplementary Fig. 1. When a chiral dopant (CB15) inducing right-handed twists is added, only (S, CW) and (L,CCW) form (see Supplementary Fig. 3 for the original images). (**e**) Example of the fluorescence profiles across the centre of the defects with *m* =  +1 with different sizes of the dark spots (dips) at *d* ~2 μm. (**f**) *d*-dependence of the dark spot sizes found for the defect with *m* =  +1. Two different sizes appear and they are comparable to *d* shown by the dashed line.

**Figure 5:**
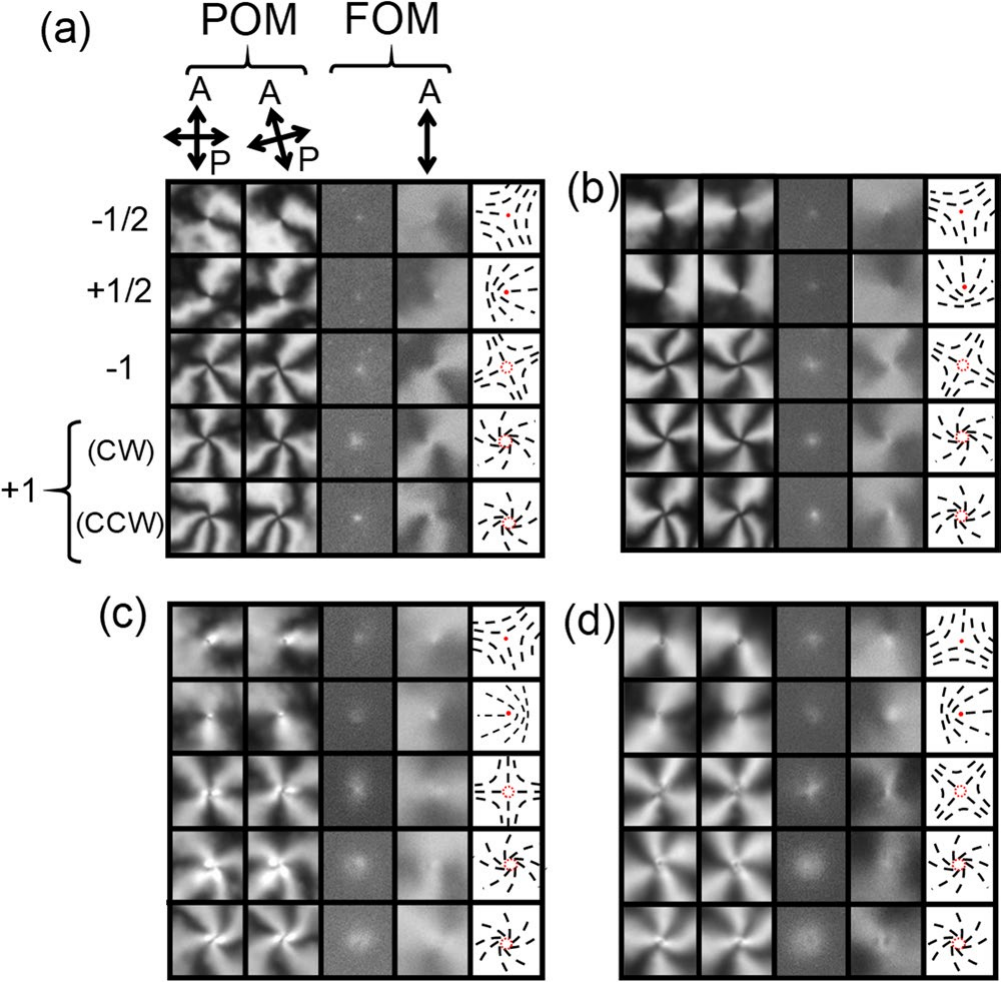
Discernible states of the defects found in schlieren textures of NLC doped with CNX. A set of microscope images (10 × 10 μm^2^ squares) of discernible defects with *m* =  ±1/2 and ±1. POM images with crossed polarisers, those with polarisers rotated by 15 degrees, FOM images and those with an analyser from left to right at *d* ~(**a**) 1, (**b**) 2, (**c**) 3, and (**d**) 4 μm. Corresponding schematics of the distribution of ***n*** projected onto the cell plane, in which black sticks represent local ***n***, are shown in each rightmost square. An example of the original images with schlieren texture is shown in Supplementary Fig. 2. In the system with CNX, clear difference in the size of the spot of the defect with *m* =  +1, which is found for the system with PMN (Fig. 4e,f), is not confirmed.

Figures 4 and 5 show all types of defects recognised in the schlieren textures of NLCs with PMN and CNX, respectively, at different thicknesses, *d*. Examples of the original images are shown in Supplementary Figs 1 and 2. The defects with *m* = ±1/2 appear as bright spots in the FOM images irrespective to the sign of the fluorescent dichroicity, suggesting the accumulation of the dye molecules at the defect cores^20,21^. Here we focus on the defects with *m* = ±1, and in FOM images they appear as dark and bright spots when the host NLC is doped with PMN and CNX, respectively. The spot size increases with increasing *d*. The results indicate that *n*_*z*_ is nonzero at the defect centre, and thus, escaped structures exist and the extent of the escaping region increases with increasing *d*. Defects with *m* = −1, appear as spots elongated in one axis in FOM images. The estimated 2D distribution of ***n*** around the defect reconstructed by images of the POM and FOM with a polariser indicates that the axis of elongation is parallel to one of the axes with radial alignment of (*n*_*x*_, *n*_*y*_).

Defects with *m* =  +1 exhibit four discernible types of patterns in the optical images of the NLC doped with PMN (Fig. 4a–d). The first difference is that dark spots with two different sizes appear at each thickness. Smaller and larger dark spots are denoted by “S” and “L”, respectively, in the brackets in Fig. 4a–d. Examples of the fluorescence profiles at *d* ~2 μm shown in Fig. 4e indicate the difference. The size of both “S” and “L” increase with increasing *d* and are comparable to *d* (Fig. 4f). On the other hand, in the optical images of the NLC doped with CNX, the size difference is not clearly recognised, although the tendency of the increase of the bright spot size with *d* is confirmed (Fig. 5). The second difference is that the streamline of (*n*_*x*_, *n*_*y*_) from the centre in the plane winds in the clockwise (CW), or counter-clockwise (CCW) manner, which is also shown in the brackets in Fig. 4a–d. It is also noted that the pure radial alignment of (*n*_*x*_, *n*_*y*_) shown in Fig. 1e is practically never found. Such curved orientation profiles as curved dark brushes in POM have been reported in previous literature^1,28–30^.

The presence of two different winding direction is indicative of chiral symmetry breaking. To confirm it, we observed the schlieren textures of a NLC doped with PMN and a small amount of CB15 that primarily induces right-handed twist distortions. Only two of the four discernible types appear for the defects with *m* =  +1 (Supplementary Fig. 3); smaller dark spots with CCW vortex (S,CCW), and larger dark spots with CW vortex (L,CW). This result indicates that the pair (S,CCW) and (L,CW) is associated with one handedness of chirality, and the other pair (S,CW) and (L,CCW) with the other handedness.

**Figure 6:**
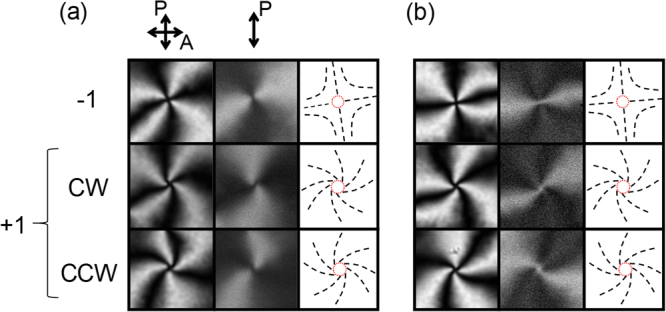
Confocal laser scanning (CLS) FOM images. Images (10 × 10 μm^2^ squares) of systems with (**a**) PMN and (**b**) CNX at *d* ~2 μm. From left to right in each panel, transmittance image with crossed-nicols-like configuration, fluorescence image, and the schematics of the 2D ***n*** distribution. Arrows with “P” indicate the polarizing direction of the incident laser and that with “A” indicates the polarising direction of the analyser. The defect centres appear as dark for a system with PMN and bright for that with CNX, which indicates the escaped structures.

We also obtained confocal laser scanning (CLS) FOM images for defects with *m* =  ±1 (Fig. 6). Since the excitation laser is linearly polarised in our setup, obtained images strongly reflect the 2D distribution of ***n***, and thus, resemble FOM ones with a polariser. Because of the circumferential contrast around the defect centre on these images, it was difficult to distinguish the difference in the size of the dark spots at the defect centre found for the defect with *m* =  +1 under FOM without the polariser. Nevertheless, the defect centres with PMN and CNX appear dark and bright, respectively, as in FOM images with a polariser, supporting the existence of the escaped structures.

## Discussions

### Structure of defects

Let us discuss the origin of the four different states found for defects with *m* =  +1. We first recall that the escape direction can be either upward or downward as shown in Fig. 1c, and that the configurations with different escape directions are of the same free energy because the two confining substrates are equivalent.

The other important contribution is that of chiral symmetry breaking as mentioned above. The chiral symmetry breaking is attributed to twist distortions due to the twist elastic constant (*K*_22_) smaller than those for splay and bend distortions (*K*_11_ and *K*_33_, respectively), and give rise to configurations with different twist sense. The up/down symmetry breaking and the chiral symmetry breaking result in four distinct configurations, shown as [U,L,CW], [U,R,CCW], [D,R,CW] and [D,L,CCW] in Fig. 1e. Here we denote the escape direction by U and D (Up/Down), the sense of the twist of ***n*** by L and R (Left-handed/Right-handed), and the sense of the spiral profile of ***n*** projected onto the cell plane by CW and CCW (ClockWise/Counter-ClockWise). It is important to note that [U,L,CW] and [D,L,CCW] are the same profile viewed from the different directions ([U,R,CCW] and [D,R,CW] are also the same). A chiral dopant inducing right-handed twist was used in the experiment shown in Supplementary Fig. 3, and therefore the profiles (L,CCW) and (S,CW) found there should be assigned to [U,R,CCW] or [D,R,CW], respectively. From the same argument for the structures with left-handed twist, [D,L,CCW] or [U,L,CW] should correspond to (S,CCW) and (L,CW). Thus the director profiles of the experimentally observed four distinct states are successfully assigned, and the size of the dark spot of the defect (S/L) is associated to the direction of the escape (U/D).

To show that this chiral symmetry breaking indeed occurs for defects with *m* =  +1, we calculate the optimised profiles of orientational order within the framework of the Landau-de Gennes formulation. The elastic energy in our Landau-de Gennes formulation corresponds to choosing *K*_22_ < *K*_11_ = *K*_33_ in the Frank elastic energy (see Methods for details). Although we do not focus on the effect of unequal *K*_11_ and *K*_33_, we note that radial far-field orientation profiles with splay distortions are realised when *K*_11_ < *K*_33_ as is the case for 5CB, and circular profiles with bend distortions when *K*_11_ > *K*_33_.

**Figure 7:**
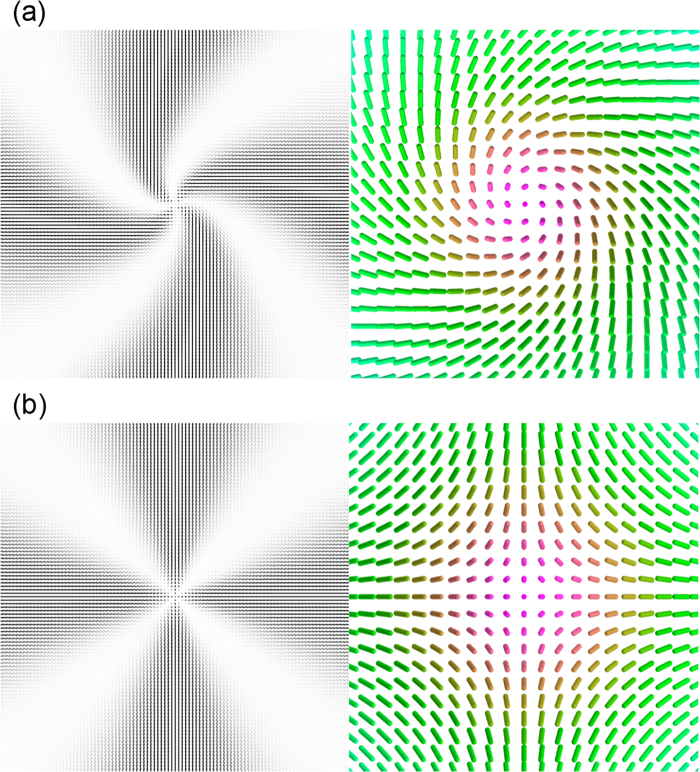
3D distribution of ***n*** around the defects with integer *m*. Calculated structures of defects with (**a**) *m* =  +1 and (**b**) *m* = −1. Here the director ***n*** is the unit eigenvector of the tensor order parameter *Q*_*ij*_ with the largest eigenvalue. The profile of ***n*** at the midplane of the cell is shown by rods. The brightness of the rod on the left is proportional to sin(2*φ*), where *φ* is the in-plane angle between the *x* (horizontal)-axis and (*n*_*x*_,*n*_*y*_). Shown on the right is a magnified view of the profile of ***n*** around the defect, where rods with the same *n*_*z*_ are depicted with the same colour. (**a**) corresponds to the state [D,R,CW] shown in Fig. 1e and (b) to [D,N,R] shown in Fig. 1d.

We relax the orientation profile from the initial profile, an ideal escaped structure without chiral symmetry breaking shown in Fig. 1e framed by a black square. Chiral symmetry breaking occurs yielding the profile shown in Fig. 7a despite the absence of randomness in the initial profile and the relaxation process, which indicates the robustness of the chiral symmetry breaking (rounding numerical errors are sufficient for the symmetry breaking) and the instability of the profiles without chiral symmetry breaking ([U,N,R] and [D,N,R] in Fig. 1e). The profile in Fig. 7a exhibits right-handed twist distortions, and that with left-handed twist distortions can emerge with equal probability because of the inversion symmetry of the free energy and the boundary conditions.

Now let us focus on the structure of a defect with *m* = −1. The projected 2D profile of a defect with *m* = −1 (Fig. 1b) appears to have C_4v_ symmetry. However, the actual 3D profile does not when the escape is appropriately taken into account (Fig. 1d)^27,35,36^; the escape is upward along one lateral axis (“Up-axis” in Fig. 1d) and downward along an axis perpendicular to the Up-axis (“Down-axis”). The symmetry of the *m* = −1 defect is therefore C_2v_, and the elongated spots observed in experiments are consistent with the C_2v_ symmetry rather than the C_4v_ symmetry. The direction of the elongation is parallel to one of the escape axes (Up-axis or Down-axis). The escaped structure shown in Fig. 1d intrinsically involves twist distortions; e.g., those with different handedness can be identified along the diagonal directions of the green square in Fig. 1d. Therefore, the defect with *m* = *−*1 cannot break the chiral symmetry in the manner that the defect with *m* =  +1 does because lower *K*_22_ would simply boost both twist distortions with different handedness locally to minimise the total energy without breaking the original C_2v_ symmetry. In Fig. 7b, we show the numerically calculated structure of a defect with *m* = −1, and the initial condition is an ideal one shown schematically in Fig. 1d. We also perform different calculations with chiral perturbations introduced in the ideal initial condition. These perturbations turned out to decay resulting in an achiral profile, which indicates the robustness of the configuration shown in Fig. 1d.

From the numerical calculations, we also find that the width of the escape remains finite, although in the case of a cylindrical capillary^1,24–26^, it is equal to the capillary radius (that is, the larger the radius, the larger the escape width). In our case of a thin cell, cell thickness *d* instead of the capillary radius enters as the characteristic length, and therefore the escape width should be proportional to *d*. This speculation is consistent with the observation in Fig. 4f that the dark spot size corresponding to the escape width increases almost linearly with *d*.

### Optics of defects

Our remaining task is to clarify why the direction of the escape (up/down) can manifest itself as the size of the dark spot. For this purpose, we investigate how light rays are bent through the LC cell near the defects with a non-uniform distribution of refractive indices^47^. The bend of light through a LC has long been known^48–50^ and applied to the LC lens technology^51^. If the lensing effect were absent, the up/down-states would be indistinguishable because the intensity of the FOM would depend only on (*n*_*x*_,*n*_*y*_) and therefore insensitive to the escape direction. Our observations motivate us to evaluate the lensing effect here.

To simplify the problem for our qualitative arguments, we consider a defect with *m* =  +1 without twist deformation shown in Fig. 1e (see Methods for the calculation detail). We restrict our discussion to rays confined in the 2D plane (*r*,*z*) that includes the *z* axis (*r* is the distance from the *z* axis). Since the director ***n*** is also confined in the (*r*,*z*) plane in our setup, the extraordinary light (where the electric field ***E*** is in the (*r*,*z*) plane) and the ordinary light (where ***E*** is perpendicular to the (*r*,*z*) plane) are decoupled, and the latter propagates without bending. Therefore, we discuss only the extraordinary component. Our calculations presented below are based on geometric optics and the principle of Fermat^5,52^.

**Figure 8:**
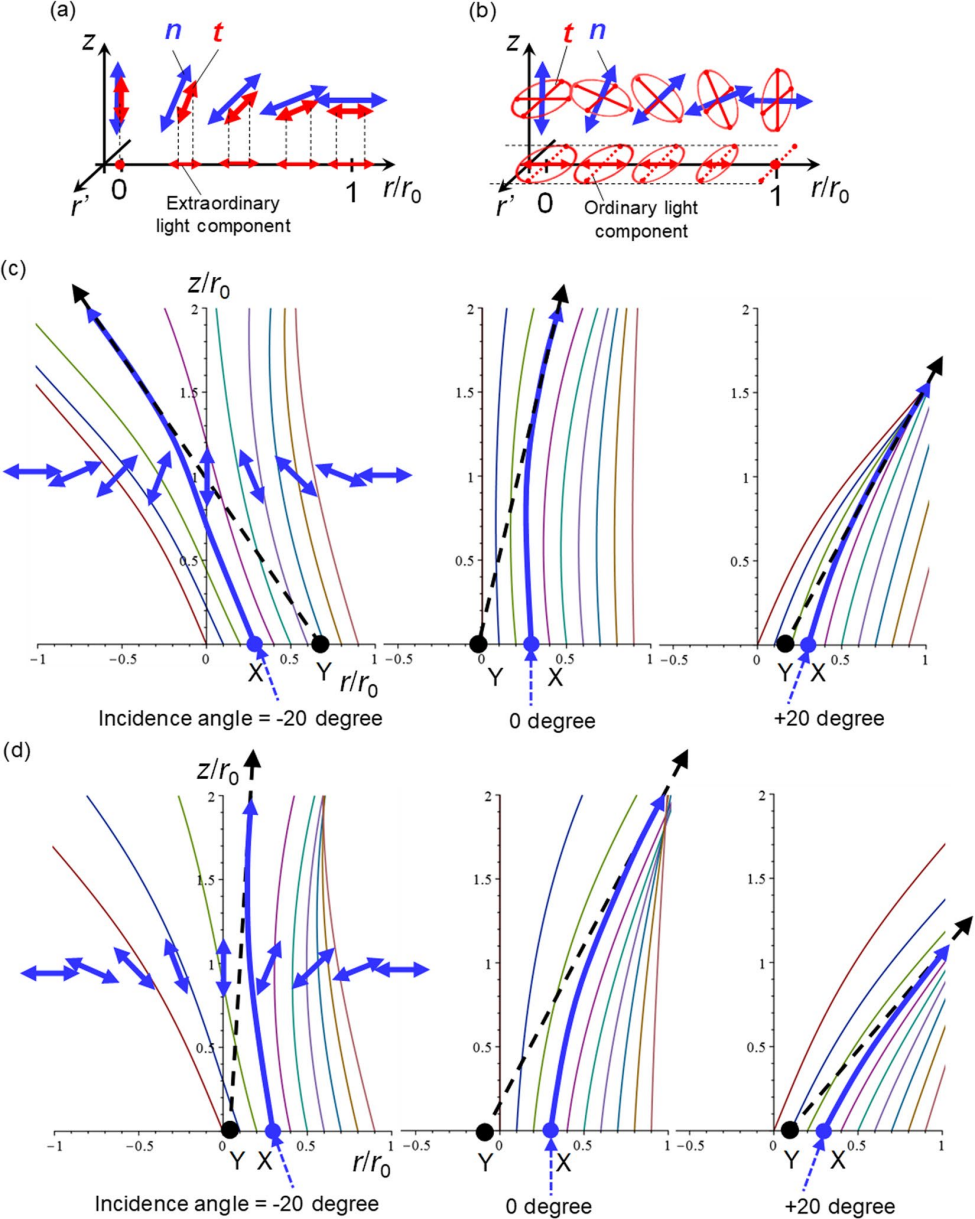
Ray-tracing around the ideal escaped structure. Schematics of the relationship between the nematic director, ***n***, and the transition dipole moment of the dye, ***t***, on a radial 2D plane with the escaped structure in the system with (**a**) PMN and (**b**) CNX. The horizontal axis indicates radial axis in the unit of *r*/*r*_0_, where *r*_0_ is the radius of the escaped region. The cases of the down-state are shown. In the system with PMN, only the extraordinary component of the excitation light and the fluorescence light is associated with the image formation. The extraordinary rays entering from the bottom side of the computational cell are shown for (**c**) the up-state and (**d**) the down-state in the *r*/*r*_0_ (horizontal axis)- *z*/*r*_0_ (vertical axis) space. Their representative ***n*** along the *r* axis are shown by double headed arrows. By considering the numerical aperture of the objective lens, the results with different incidence angles of rays, −20, 0, and + 20 degrees, are shown to qualitatively capture the bending characteristics. The light from the point X seems to originate at the point Y due to the LC lensing effect (Incident rays entering at *r* = 0.3 are highlighted by arrows as examples), suggesting that the original fluorescence distribution shrinks toward the centre of the defect in the resulting images. This lensing effect is more remarkable for the down-state as shown in (**d**).

The transition dipole moment ***t*** of PMN on average aligns along ***n*** (Fig. 2b), and thus, the extraordinary component dominates both excitation and emitted fluorescence rays (Fig. 8a). On the other hand, ***t*** is distributed perpendicularly to ***n*** for CNX (Fig. 2c). Therefore, excitation and emitted fluorescence rays contain both the ordinary and the extraordinary components depending on |*n*_*z*_|; the extraordinary component grows as |*n*_*z*_| varies from 0 (a peripheral part of the defect) to 1 (the defect centre) (Fig. 8b). This simply suggests that the systems with PMN and CNX show different lensing effects; a clearer effect is expected for the system with PMN than that with CNX, in agreement with the experimental result that the up/down-states are indistinguishable from the microscopy images for the latter. The following discussion on extraordinary rays therefore concerns microscope images on the system with PMN.

The calculated rays entering from different points near the defect centre at the bottom side of the cell with different incident angles are shown for both up/down-states in Fig. 8c,d. Although both states can bend the rays, the incident rays clearly tend to deviate more from the defect centre for the down-state. In the following, we discuss qualitatively how the bending of rays modulates 2D distributions of the excitation intensity and distort the resulting fluorescence images.

The excitation rays entering from the top side of the cell with the down- (or up-) state roughly correspond to those entering from the bottom side of the cell with the up- (or down-) state shown in Figs 8c (or 8d). Thus, for the down-state the excitation intensity is only slightly blurred and almost homogeneous. For the up-state, the excitation rays deviate toward the radial direction as rays travel deeper into the cell, resulting in the decreased intensity of excitation at the centre of the defect and the increased intensity roughly at the peripheral parts of the escaped region (*r*/*r*_0_ ~1, where *r*_0_ is the radius of the escaped region), in which the number density of the bent rays is highest at *z*/*r*_0_ = 1~2 in Fig. 8d. Since the emitted fluorescence rays from the up-state propagate with the little bending following the traces shown in Fig. 8c, the resulting fluorescence image of the defect is expected to be a simple manifestation of the actual profile of |*n*_*z*_| in the radial direction with slightly increased intensity at the peripheral position.

On the other hand, the emitted fluorescence rays from the down-state, which has been excited almost homogeneously, bend toward the radial direction as shown in Fig. 8d and the deviation becomes larger for rays traveling from deeper parts. The ray from the point X at the vicinity of the defect centre in Fig. 8d bends in the radial direction, and thus, seems to originate at the point Y which is deviated from X towards the defect centre. As a result, the fluorescence intensity distribution is expected to be modulated and distorted so that the escaped region in the resulting fluorescence image appears as a smaller dark region. These qualitative arguments agree well with the experimental results, in which the dark region of the down-state appear smaller than that of the up-state and the fluorescence intensity in the peripheral region surrounding the dark spot is larger than that at a large distance from the defect (Fig. 4).

The same argument applies to the identification of the up- and down-axes of a defect with *m* = −1 (Fig. 1d) from the FOM images in Fig. 4; the direction of the elongation of the dark spot indicated by yellow arrows in Fig. 4 corresponds to the up-axis. Also in the system with CNX, although they are not so clear as the system with PMN, some of the bright spots in the FOM images of defects with *m* = *−*1 at larger *d* show elongated shapes (Fig. 5), in which the elongated direction should correspond to the up-axis.

Overall, the systematic modulations of the defect images due to the lensing effect are critical for the distinction of the up/down-states. For more precise discussions, rigorous optical treatment based on the solution of the full Maxwell equations will be required instead of the geometrical optics and the ray-tracing method^53^, which remains to be studied in future.

In summary, using a conventional optical microscope, we have uncovered the hidden states of the defects with integer strength, *m*, in the classical schlieren textures. For defects with *m* = +1, four states are identified as the result of up-down and chiral symmetry breaking. For those with *m* = −1, the directions of the two characteristic axes are distinguished. Two types of fluorescent dyes with different alignments of the transition dipole moment with respect to the local nematic director ***n*** enable us to confirm the escaped structures of defects with integer *m*. Through the Landau-de Gennes numerical modelling, we have calculated their detailed escaped structures and associated them with the observed different states. The simulation results also suggest that the chiral symmetry-broken structures showing 2D spiral-like streamline of ***n*** emerge only for defects with *m* =  +1 due to the lower elastic constant for the twist deformation than those of splay and bend ones, in agreement with the experimental results. Moreover, we have attributed the fluorescence images modulated depending on the escaped direction of ***n*** to the LC lensing effect due to the characteristic 3D distribution of the refractive indices around the defect. This suggests the importance of considering the lensing effect on the image analysis of more complex LC defects, e.g., defects with higher values of *m* (refs^15,54^), to reveal their hidden structural details. Clarification of the detailed structures of various defects in LCs could reveal their potential as unique templates for micro-patterning of asymmetric and/or chiral objects ranging from small molecules to colloidal particles. Since the escaped structures of the defects with *m* =  ±1 can locally and systematically modulate light depending on the size and the states, they also have potential as micro-light-modulators for gradient-index micro-lenses and a fine maskless direct patterning technique in photo-lithography.

## Methods

### Materials

A NLC, 4-cyano-4′-pentylbiphenyl (5CB), whose twist elastic constant is significantly lower than those for splay and bend distortions^55^, was purchased from Wako Chemical. A fluorescent dye pyrromethene 597 (PMN, [[(4-*tert*-Butyl-3,5-dimethyl-1*H*-pyrrol-2-yl)(4-*tert*-butyl-3,5-dimethyl-2*H*-pyrrol-2-ylidene)methyl]methane](difluoroborane) was purchased from Sigma-Aldrich. Another fluorescent dye C-Naphox (CNX, racemic mixture) was synthesized according to the method previously reported by some of the authors^37^. Poly-amic acid (Poly(pyromellitic dianhydride-co-4,4′-oxydianiline), and *N*-methylpyroridone (NMP) for a precursor solution used for polyimide coating with planar alignment were obtained from Sigma-Aldrich. As a right-handed chiral reagent, 4-cyano-4′-((*S*)-2-methylbutyl)biphenyl (CB15, Merck) was used to investigate the effect of chirality.

### Preparation of planar degenerate LC cells

We adopted a wedge cell with planar alignment without preferential direction (degenerate planar alignment) to observe very thin regions at the thickness of roughly 1 to 4 μm. A spin-coater (MSA-150, Mikasa) was used at 5,000 rpm for 60 s to coat cover glass slides with a poly-amic acid solution (1 wt%). The coated slides were then heated on a hot plate at 70 °C for 2 min to evaporate the solvent, and subsequently placed in an oven at 180 °C for 3 h to induce the transformation of poly(amic acid) to polyimide through the dehydration reaction. Then, two slides were assembled to a wedge cell using a Kapton^®^ spacer placed at one side of the cell with a small amount of adhesive (liquid gasket1212, ThreeBond). The slides were held together using clips during curing of the adhesive. Although the wedge angle was ideally ~0.036 degree, the actual cell showed spatial fluctuation of the wedge angle (and the cell gap), probably due to the bending of the cover glass and inhomogeneity in the thickness of the polyimide layer. The NLC, 5CB, doped with 0.01 wt% PMN or 0.05 wt% CNX was injected into the wedge cell at an elevated temperature (40~45 °C) via capillary action. The sample was cooled to room temperature (22 ± 2 °C) and used for microscopic observations. Samples doped with CB15 at ~0.2 wt%, which gives the cholesteric pitch much larger than *d*, were prepared employing the same procedure.

For measurements of fluorescence dichroic properties, a planar cell with unidirectional planar alignment (KSRP-02/A111P1NSS05, EHC; cell gap of 2 ± 0.5 μm) filled with 5CB doped with dyes was used.

### Optical microscopy

We observed the LC alignment using a transmitted polarizing optical microscope (POM) (BX-51P, Olympus) under crossed-nicols conditions.

To observe fluorescent images, we used a conventional fluorescence optical microscope (FOM), where the light source was mounted above the sample and the excitation light passed through the microscope objective lens on its way toward the sample. A Xe lamp (75 W) was used as the light source. To detect the emission from PMN and CNX, we used a fluorescence filter set (U-MWIB-3, Olympus) comprising an excitation filter that transmitted light with wavelengths between 460 nm and 495 nm, and an emission filter that transmitted light with wavelength larger than 510 nm. The images were collected at the pixel size of 0.154 μm using a Nikon DS-Qi1-Mc CCD (charge-coupled device) camera connected to a computer and controlled through imaging software (NIS-Elements, Nikon). An objective lens with a numerical aperture (NA) of 0.95 (UPLSAPO40 × 2, Olympus) was mainly used.

For the measurements of dichroic properties, the fluorescence intensity was measured using the FOM with a polarizer (analyser), through which the emitted fluorescence passed. The average intensities were measured at different angles, *Φ*, between the axis of the polarizer and the nematic director of the cell. When the maximum and minimum intensities were at *Φ* = 0 degree and ±90 degree, respectively, the transition moment of dye, ***t***, was aligned to the nematic director, ***n***, and the dye had positive dichroic fluorescence property. When the intensity variation behaves in an opposite manner, ***t*** was perpendicularly aligned to ***n***, and the dye had negative dichroic fluorescence property. To further confirm the alignment of dyes in 5CB, the same measurements were conducted under the AC voltage to align ***n*** along the optical axis, that is, perpendicular to the cell.

We obtained microscope images in four regions exhibiting bluish white, yellow, blue, and light green colours in the POM under the crossed-nicols condition. Their thicknesses were estimated to be approximately 1, 2, 3, and 4 μm (error ± 0.5 μm) using the Michel-Levy interference chart [see, e.g., Olympus Microscopy Resource Centre homepage] with the birefringence of 5CB being ~0.18 at 23 °C^56^. For image acquisition, the focus was carefully adjusted to give tack-sharp peaks of defects with *m* =  ±1/2 (ref.^20^). The contrast of fluorescence images was linearly enhanced to maximize the visibility.

A confocal laser scanning (CLS) FOM (A1^+^ system, Nikon) was used to obtain the fluorescence images of defects with integer *m*. An optically pumped semiconductor laser (LU-N4 Laser Unit, Nikon, equipped with Sapphire 488, Coherent Inc.) was used to excite the fluorescent molecules at 488 nm and the emitted light between 525 and 595 nm was collected. An objective lens with an NA of 1.45 (PlanApoTIRF60 ×, oil, Nikon) was used. The CLS-FOM images were acquired at the pixel size of typically 60(*x*) × 60(*y*) nm^2^. The excitation laser was linearly polarized in *y* direction and all emitted fluorescence was collected.

### Landau-de Gennes numerical modelling

A second rank tensor (*Q*_*ij*_) is used for the orientational order parameter, and the free energy of the system is formally expressed as

1 $$F=\int dx\,dy[{\int }_{0}^{d}dz\,\{\,{f}_{{\rm{l}}{\rm{o}}{\rm{c}}{\rm{a}}{\rm{l}}}+{f}_{{\rm{g}}{\rm{r}}{\rm{a}}{\rm{d}}}\}+{f}_{{\rm{s}}0}+{f}_{{\rm{s}}d}],$$

where *d* is the thickness of the cell and *z* is the coordinate along the cell normal. The bulk parts of the free energy density read^15^

2 $${f}_{{\rm{local}}}=\frac{1}{2}A{\rm{Tr}}\,{Q}^{2}-\frac{1}{3}B\,{\rm{Tr}}{Q}^{3}+\frac{1}{4}C{({\rm{Tr}}{Q}^{2})}^{2},$$

3 $${f}_{{\rm{g}}{\rm{r}}{\rm{a}}{\rm{d}}}=\frac{1}{2}{L}_{1}{({\rm{\nabla }}\times Q)}_{ij}{({\rm{\nabla }}\times Q)}_{ij}+\frac{1}{2}{L}_{2}{({\rm{\nabla }}\cdot Q)}_{j}{({\rm{\nabla }}\cdot Q)}_{j},$$

where summations over repeated indices are implied, ${({\nabla }\times Q)}_{ij}\equiv {{\epsilon }}_{ist}{{\nabla }}_{s}{Q}_{tj}$, and (*∇* ⋅ *Q*)_*j*_ ≡ *∇*_*i*_ *Q*_*ij*_, where ${{\epsilon }}_{ist}$ is the Levi-Civita antisymmetric symbol. We used typical material parameters for 5CB (ref.^57^); *A* = 0.044 × 10^6^(*T* − *T* ^*^) J m^−3^ K^−1^ with *T* ^*^ = 307 K and *T* = 296 K (room temperature), *B* = 0.816 × 10^6^ J m^−3^, *C* = 0.45 × 10^6^ J m^−3^, *L*_1_ = 6 × 10^−12^ J m^−1^ and *L*_2_ = 18 × 10^−12^ J m^−1^. Note that when a uniaxial form of the order parameter, *Q*_*ij*_ = *Q*_0_(*n*_*i*_ *n*_*j*_ − (1/3)*δ*_*ij*_), is assumed, *f*_grad_ is reduced to the Frank elastic energy with *K*_11_=*K*_33_=(*L*_1_ + *L*_2_)*Q*_0_^2^ and *K*_22_ = 2*L*_1_ *Q*_0_^2^. Therefore, our choice of *L*_1_ and *L*_2_ yields *K*_22_ smaller than *K*_11_(=*K*_33_) as is the case for 5CB.

The surface free energy density for two confining surfaces, *f*_s0_ and *f*_s*d*_ were taken to be identical in our calculations, and we adopted the following form commonly used to model surfaces with planar degenerate anchoring^58^:

4 $${f}_{s0}={f}_{sd}=\frac{1}{2}{W}_{1}{\rm{Tr}}{(\tilde{Q}-{\tilde{Q}}^{\perp })}^{2}+\frac{1}{2}{W}_{2}{({\rm{Tr}}{\tilde{Q}}^{2}-{Q}_{{\rm{s}}}^{2})}^{2},$$

where *Q*_s_ defines the strength of orientational order at the surface ${\tilde{Q}}_{ij}\equiv {Q}_{ij}+(1/3){Q}_{{\rm{s}}}{\delta }_{ij}$, and ${\tilde{Q}}_{ij}^{\perp }\equiv {P}_{ik}{\tilde{Q}}_{kl}{P}_{lj}$ with *P*_*ij*_ = *δ*_*ij*_ − *ν*_*i*_ *ν*_*j*_ being the projection operator where ***ν*** is a unit vector normal to the surface. We used *W*_1_ = *W*_2_ = 10^−4^ J m^−2^, a typical value for moderate anchoring strength.

The above-mentioned free-energy functional was minimised on a 201 × 201 × 51 cubic lattice with the lattice constant being $\Delta x\simeq 20$ nm (Therefore the cell thickness is approximately 1 μm). The minimisation was carried out by solving a simple rotational relaxation equation ∂*Q*_*ij*_ $({\boldsymbol{r}})$/∂*t* = −*δF*/*δQ*_*ij*_ $({\boldsymbol{r}})$ + *λδ*_*ij*_, where the Lagrange multiplier *λ* ensures Tr*Q* = 0.

The initial profile with an “escape” for *m* = +1 was set to be uniform along the *z* direction, and taken to be ${Q}_{ij}(x,y,z)={Q}_{{\rm{i}}{\rm{n}}{\rm{i}}{\rm{t}}}({n}_{i}(x,y){n}_{j}(x,y)-(1/3){\delta }_{ij})$, where the director ***n***(*x*,*y*) was set to the following:

5 $${\boldsymbol{n}}=\{\begin{array}{cc}\sin (\theta (r)){{\boldsymbol{e}}}_{r}+\,\cos (\theta (r)){{\boldsymbol{e}}}_{z} & r\le {r}_{0}\\ {{\boldsymbol{e}}}_{r} & {\rm{o}}{\rm{t}}{\rm{h}}{\rm{e}}{\rm{r}}{\rm{w}}{\rm{i}}{\rm{s}}{\rm{e}}\end{array}\,$$

where *Q*_init_ = 1, $r=\sqrt{{(x-{x}_{c})}^{2}+{(y-{y}_{c})}^{2}}$ with (*x*_*c*_, *y*_*c*_) being the coordinates of the centre of the cell, *r*_0_ = 10Δ*x* is the radius of the escape, ***e***_*r*_ = (*x* − *x*_*c*_,*y* − *y*_*c*_,0)/*r* is a radial unit vector, with and ***e***_*z*_ is a unit vector along the *z* direction. *θ*(*r*) = 2tan^−1^(*r*/*r*_*c*_) is the profile of escape obtained analytically for the “one-constant” case^1,24,25^, and this choice of *θ*(*r*) yields the down-state. We fixed ***n*** to ***e***_*r*_ at the outer boundaries in the (*x*,*y*) plane. Although this initial profile is achiral, it turned out that rounding errors were enough to induce the transition to a chiral state mentioned in the main text.

The initial profile for *m* = −1 is the same as that for *m* = +1, with ***n*** being flipped about the *x* axis locally. We performed another calculation with chiral perturbations introduced in the above-mentioned initial profile for *m* = −1, and these perturbations turned out to decay, resulting in an achiral profile mentioned in the main text.

### Ray-tracing simulation

As noted in the main text, the main focus of our ray-tracing calculations is to provide a qualitative argument on the appearance of the escaped defects. Therefore, we consider a simple case of achiral configuration of the orientational order given in eq. (5) for *r* ≤ *r*_0_, in which the director ***n*** lies in the (***e***_*r*_, ***e***_*z*_) plane, and so does the light ray of interest. Now *θ* (*r*) = ± 2tan^−1^ (*r*/*r*_0_), where + and − are for the down state and the up state, respectively.

According to Fermat’s principle, the path of extraordinary light from point A to B minimises the following integral^5^:

6 $$F={\int }_{\,A}^{\,B}dl\,\sqrt{{n}_{\,{\rm{o}}}^{2}{\cos }^{2}\varphi +{n}_{\,e}^{2}{\sin }^{2}\varphi }$$

where *dl* is the line element of the path, *n*_o_ and *n*_e_ are the ordinary and the extraordinary refractive indices, respectively, and *ϕ* is the angle between the tangent of the path and ***n***. In our calculation, we set *n*_e_ = 1.7 and *n*_o_ = 1.5, typical values for 5CB. When we denote the path by *r*(*z*), $dl=dz\sqrt{1+{(dr/dz)}^{2}}$. The unit vector tangent to the path is ${\boldsymbol{t}}=(dr/dz,1)/\sqrt{1+{(dr/dz)}^{2}}$, and therefore $\cos \,\varphi ={\boldsymbol{n}}\cdot {\boldsymbol{t}}=(\sin \,\theta (r(z))\,dr/dz+\,\cos \,\theta (r(z)))/\sqrt{1+{(dr/dz)}^{2}}$. The integral to be minimised is finally written as $F={\int }_{\,A}^{\,B}dz\,f\{r(z),dr/dz\}$ with

7 $$f\{r(z),dr/dz\}=\sqrt{(1+{(\frac{dr}{dz})}^{2}){n}_{\,e}^{2}-({n}_{\,e}^{2}-{n}_{\,o}^{2}){(\sin \theta (r(z))\frac{dr}{dz}+\cos \theta (r(z)))}^{2}}$$

The Euler-Lagrangian equation ∂*f*/∂*r* − (*d*/*dz*)(∂*f*/∂(*dr*/*dz*)) = 0 was solved numerically with the aid of Maple 2016.2 for given *r*(0). An additional initial condition must be given for *dr*/*dz*|_*z* = 0_, which is determined by appropriately taken into account the refraction at the interface (*z* = 0) between the liquid crystal and the outer medium (whose refractive index was set to 1.5).

## Electronic supplementary material

SI-file
