# Supplementary material for: Uncovering different states of topological defects in schlieren textures of a nematic liquid crystal
Source: Sci Rep. 2017 Dec 1;7:16814. doi: 10.1038/s41598-017-16967-1 (PMC5711923; doi:10.1038/s41598-017-16967-1)
Supplement: Supplementary file 1 — SI-file [file 41598_2017_16967_MOESM1_ESM.pdf]

Supporting Information *for* Uncovering different states of topological defects in schlieren textures of a nematic liquid crystal

Takuya Ohzono[1]*, Kaoru Katoh[2], Chenguang Wang[3], Aiko Fukazawa[4], Shigehiro Yamaguchi[3,4], Jun-ichi Fukuda[5]*

[1]*Research Institute for Sustainable Chemistry, National Institute of Advanced Industrial Science and Technology (AIST) 1-1-1 Higashi, Tsukuba 305-8565, Japan*. *E-mail: ohzono-takuya@aist.go.jp

[2]*Biomedical Research Institute, AIST, 1-1-1 Higashi, Tsukuba 305-8566, Japan*.

[3]*Institute of Transformative Bio-Molecules (ITbM), Nagoya University, Furo, Chikusa, Nagoya 464-8602, Japan.*

[4]*Department of Chemistry, Graduate School of Science Nagoya University, Furo, Chikusa, Nagoya 464-8602, Japan.*

[5]*Department of Physics, Kyushu University, 744 Motooka, Nishi-ku, Fukuoka 819-0395, Japan.*

List of contents:

Supplementary Fig. 1 Page 2
Supplementary Fig. 2 Page 3
Supplementary Fig. 3 Page 4

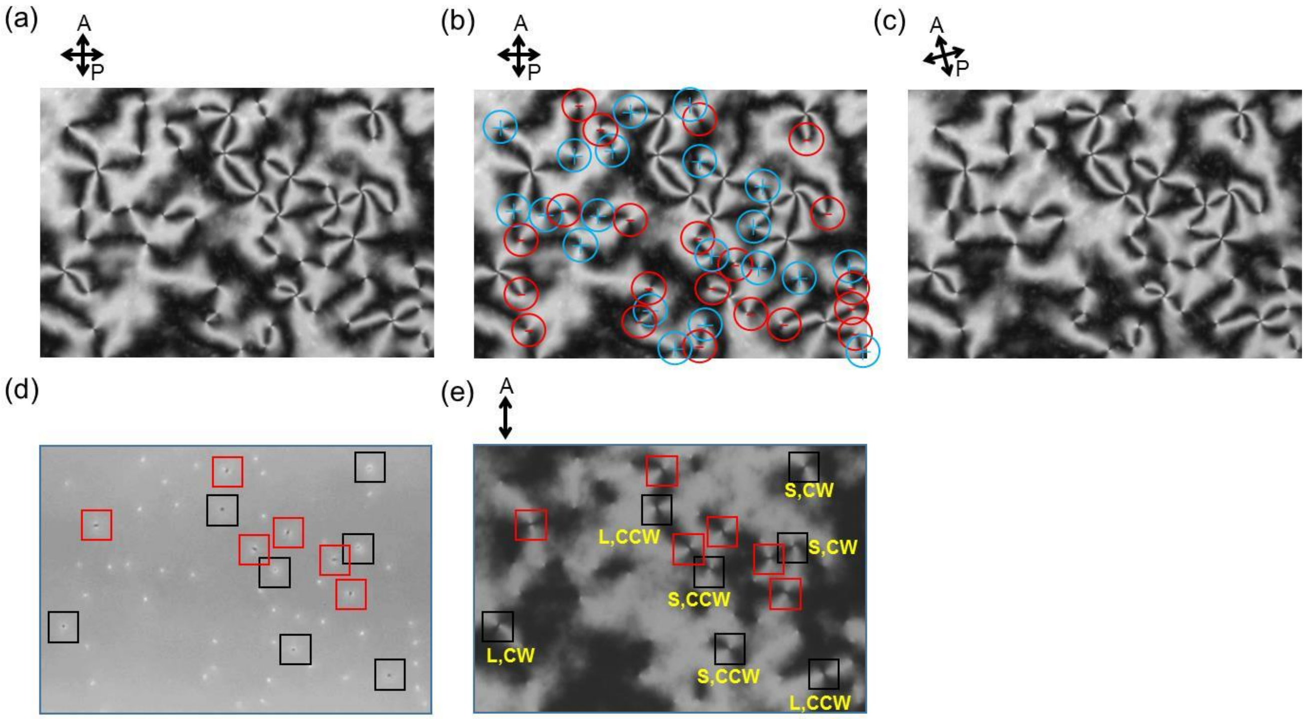

Supplementary Figure 1. A typical set of microscopy images of NLC doped with PMN at *d* ~ 2 μm. (a) (b) and (c) POM images with crossed nicols. (d) FOM image. (e) FOM image with an analyser in the *y* direction. Size of each image: 140μm×90μm. In (b), defects with *m* = -1/2 and +1/2 are highlighted by red and blue circles, respectively. In (d) and (e), defects with *m* = -1 and +1 are highlighted by red and black squares, respectively. Defects with *m* = +1 in FOM image appear as smaller (S) or larger (L) dark spots. Moreover, they show two types of spiral-like streamline of ***n*** from the centre in the plane [ClockWise (CW), or Couter-ClockWise (CCW) spiral]. Four types of defects with *m* = +1 are marked with yellow characters.

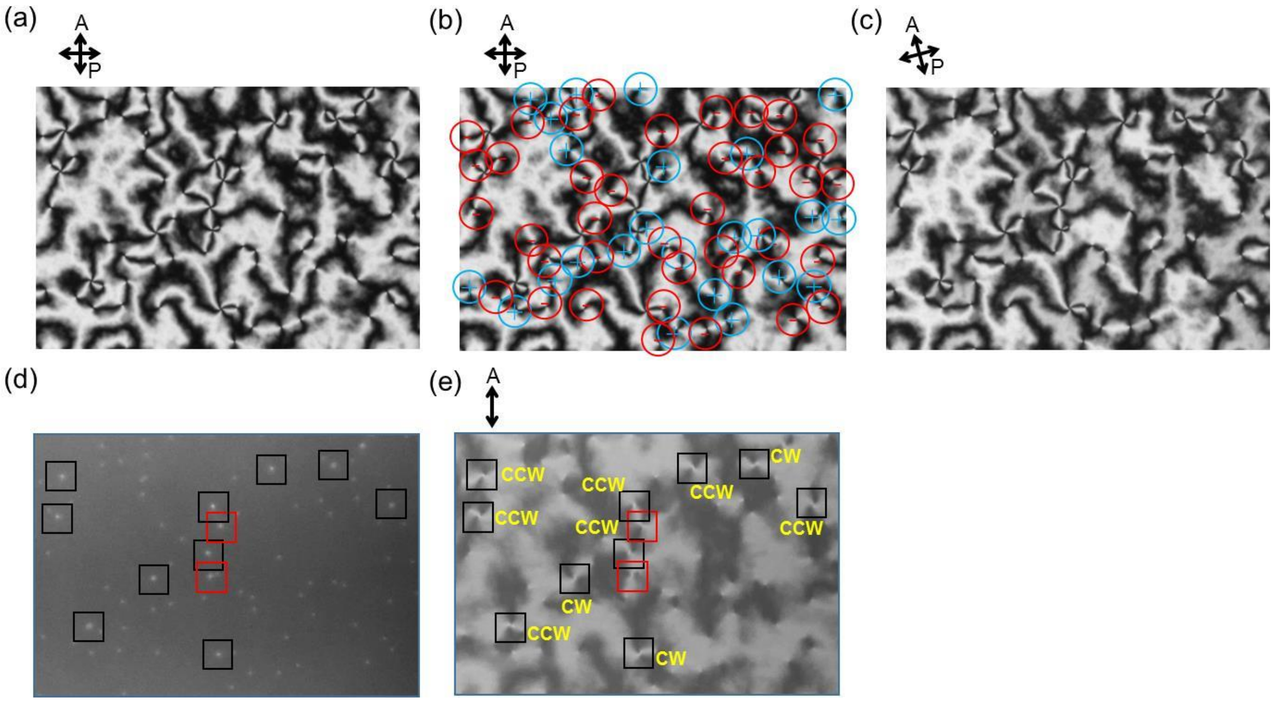

Supplementary Figure 2. A typical set of microscopy images of NLC doped with CNX at *d* ~ 2 μm. (a), (b) and (c) POM images with crossed nicols. (d) FOM image. (e) FOM image with an analyser in the *y* direction. Size of each image: 140μm×90μm. In (b), defects with *m* = -1/2 and +1/2 are highlighted by red and blue circles, respectively. In (d) and (e), defects with *m* = -1 and +1 are highlighted by red and black squares, respectively. The difference in the dark spot size for defects with *m* = +1 in FOM image can hardly be recognized in contrast to the case with PMN. On the other hand, they show two types of spiral-like streamline of ***n*** from the centre in the plane [ClockWise (CW), or Couter-ClockWise (CCW) spiral]. Two types of defects with *m* = +1 are marked with yellow characters.

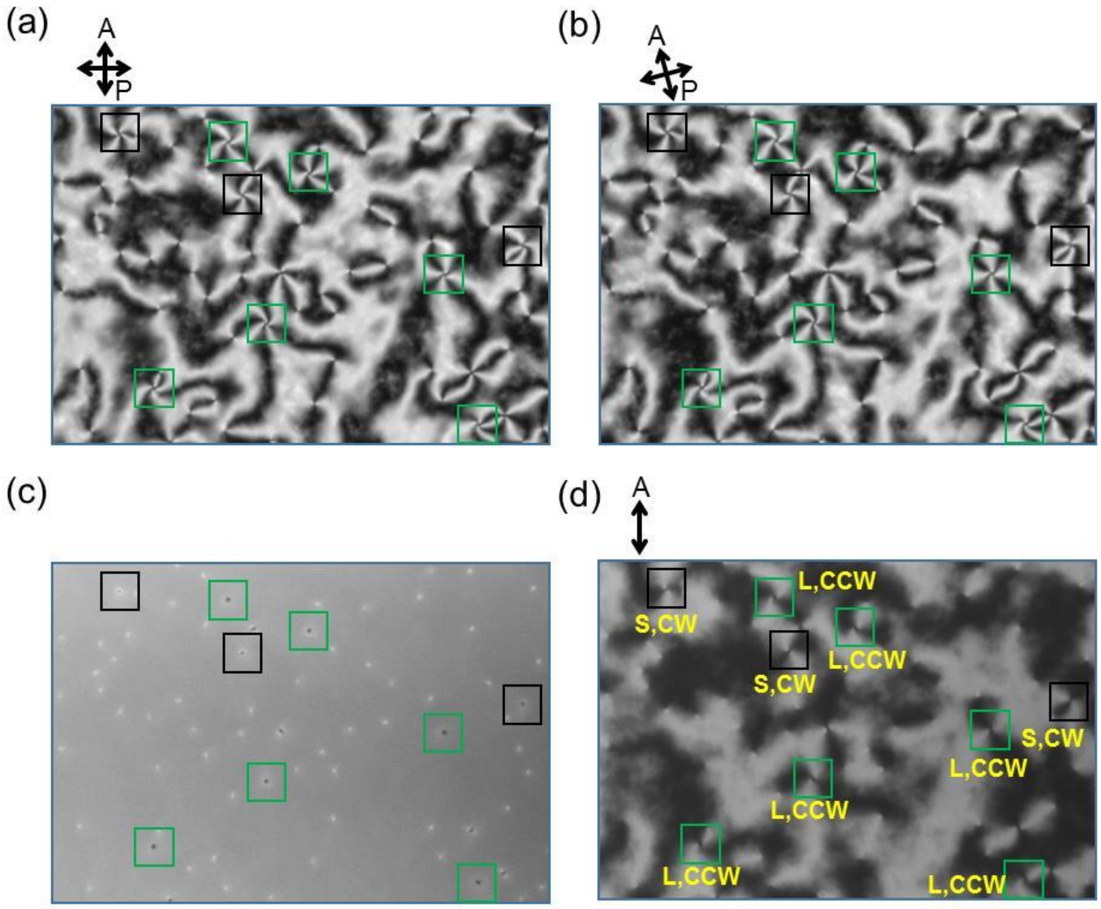

Supplementary Figure 3. A typical set of microscopy images of NLC doped with PMN and CB15 at d ~ 2 μm. (a) and (b) POM images with crossed nicols. (c) FOM image. (d) FOM image with an analyser in the y direction. Size of each image: 140μm×90μm. Defects with m = +1 are highlighted by squares. Defects with m = +1 in FOM image appear as smaller (S) or larger (L) dark spots. Moreover, they show two types of spiral-like streamline of $\boldsymbol{n}$ from the centre in the plane [ClockWise (CW), or Couter-ClockWise (CCW) spiral]. Note that only two ([S,CW] and [L,CCW], highlighted by black and green squares, respectively) out of four possible types of defects (Supplementary Fig. 1) are recognized and marked with yellow characters. This result has been confirmed with no exception by assessing 39 defects with m = +1 over the region of 112,493 μm^2.
